# Supplementary material for: Simultaneous multi-signal quantification for highly precise serodiagnosis utilizing a rationally constructed platform
Source: Nat Commun. 2019 Nov 25;10:5361. doi: 10.1038/s41467-019-13358-0 (PMC6877524; doi:10.1038/s41467-019-13358-0)
Supplement: Supplementary file 1 — Supplementary information [file 41467_2019_13358_MOESM1_ESM.pdf]

Supplementary information

Yuxin Liu *et al.* Simultaneous Multi-signal Quantification for Highly Precise Serodiagnosis Utilizing Rationally Constructed Platform

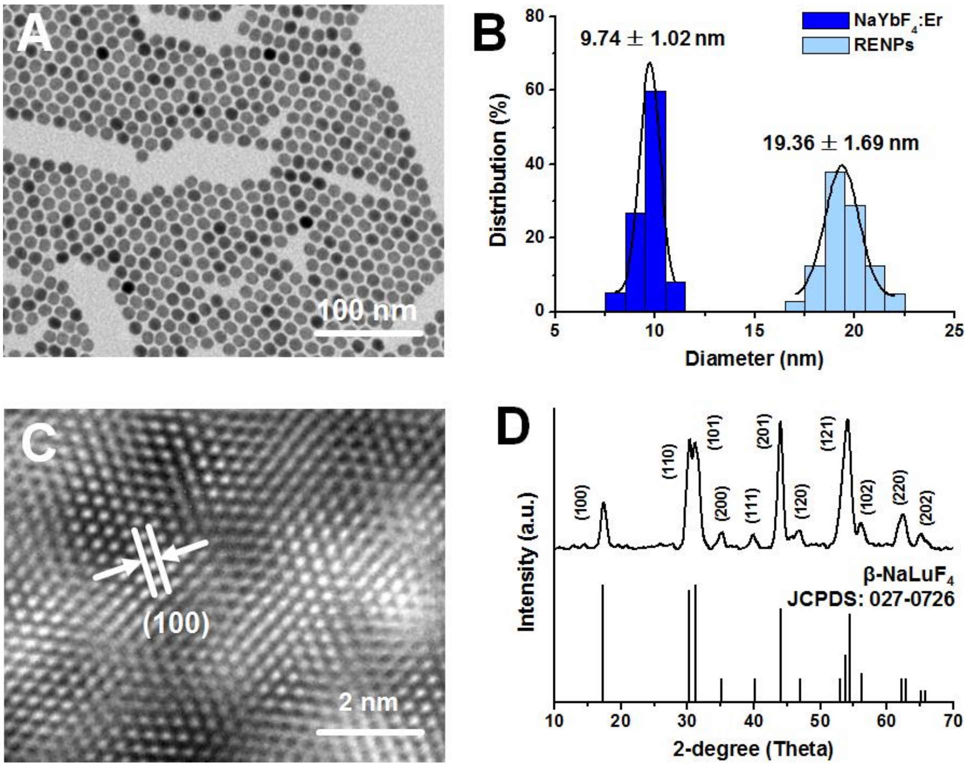

Supplementary Figure 1. Extra physical characterization of $NaYbF_4$:Er and RENPs. A) transmission electron microscopic image of $NaYbF_4$:Er. B) Diameter distribution of $NaYbF_4$:Er and RENPs. C) Fast F öurier-transform-enhanced high-resolution transmission electron microscopic image of RENPs. D) Powder X-ray diffraction pattern of RENPs. The standard card of β-$NaLuF_4$ was used for comparison (JCPDS: 027-0726).

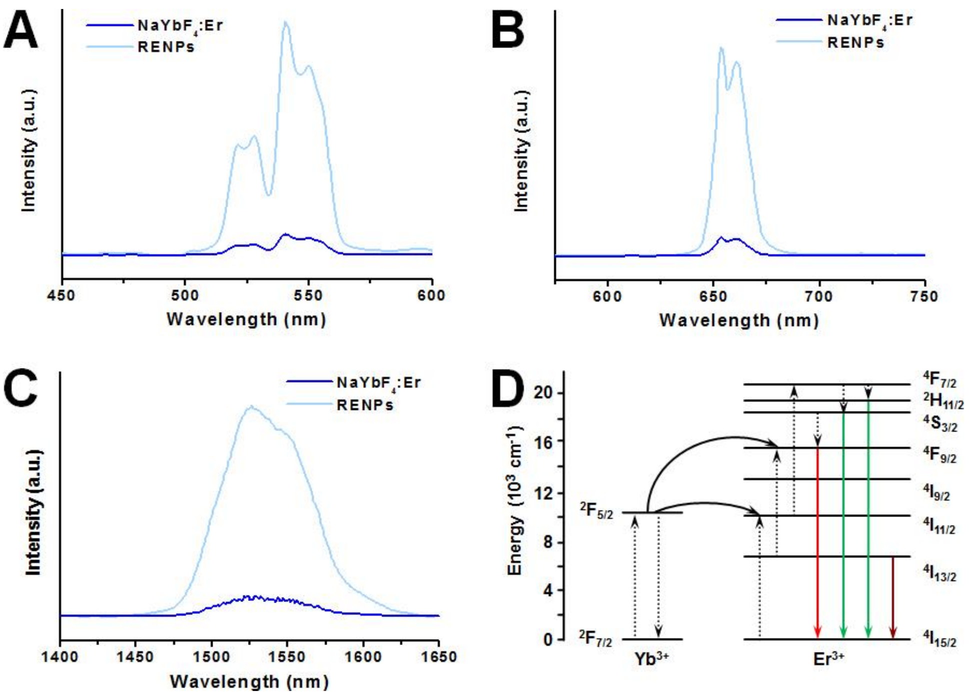

Supplementary Figure 2. Luminescence properties of $NaYbF_4$:Er and RENPs. Green UCL A), red UCL B), and SWIR luminescence C) spectra of $NaYbF_4$:Er and RENPs. D) Proposed energy transfer processes responsible for the UCL and SWIR luminescence of RENPs. Dotted arrows represent non-radiative transitions and curved arrows represent nonradiative energy transfer. Green, red and wine arrows represent green UCL, red UCL, and SWIR luminescence, respectively.

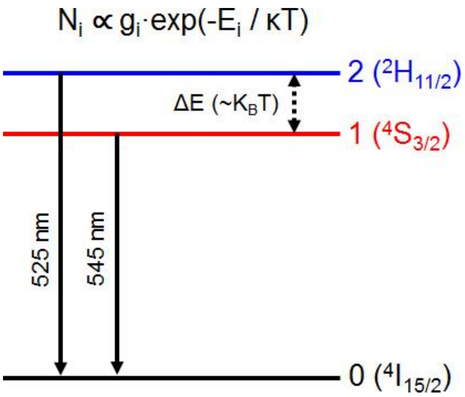

Supplementary Figure 3. Mechanism of temperature-responsive luminescence of $NaYbF_4$:Er. The energy level diagram of the green UCL emissions for the $NaYbF_4$:Er by a 980 nm laser excitation.

The population of energy levels $^2H_{11/2}$ and $^2S_{3/2}$ were corresponded to Boltzmann distribution. Therefore, the $^2H_{11/2} \rightarrow {}^4I_{15/2}$ (525 nm) and $^2S_{3/2} \rightarrow {}^4I_{15/2}$ (545 nm) transitions were thermo-sensitive due to the thermal induced re-distributions in the populations of energy levels.

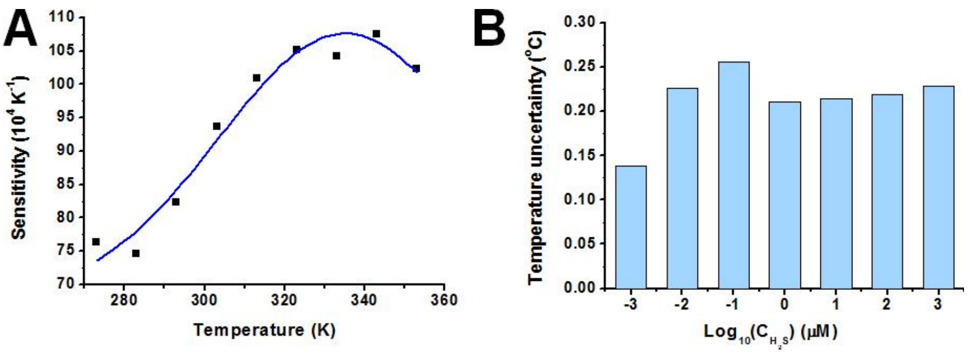

Supplementary Figure 4. Thermo-sensitive capacities of RENPs. Thermal sensitivity A) and temperature uncertainty B) of RENPs-based luminescent nanothermometer.

The thermal sensitivity of RENPs was calculated from experimental data included in Fig. 1D by a standard equation for ratiometric luminescent nanothermometers:

$$S = \frac{1}{R}\frac{dR}{dT} \qquad (1)$$

where S is thermal sensitivity, R is I_{525}/I_{545} luminescence ratio and T is temperature in Kelvin unit. The thermal sensitivity of RENPs was calculated to be 0.0108 K^{-1}, which was higher than some of typical luminescent nanothermometers and comparable to most rare-earth-based nanothermometers (Supplementary Table 1).

The temperature uncertainty of RENPs was calculated from experimental data included in Fig. 2G by an equation in first order approximation:

$$\delta T = \frac{1}{S}\frac{\Delta I}{I} \qquad (2)$$

where δT is temperature uncertainty, S is thermal sensitivity, ΔI is background noise of luminescence spectrum and I is luminescence intensity. The temperature uncertainty of RENPs was calculated to be 0.2559 K, which was less than 1 K and hence applicable to quantify temperature according to previous reports.

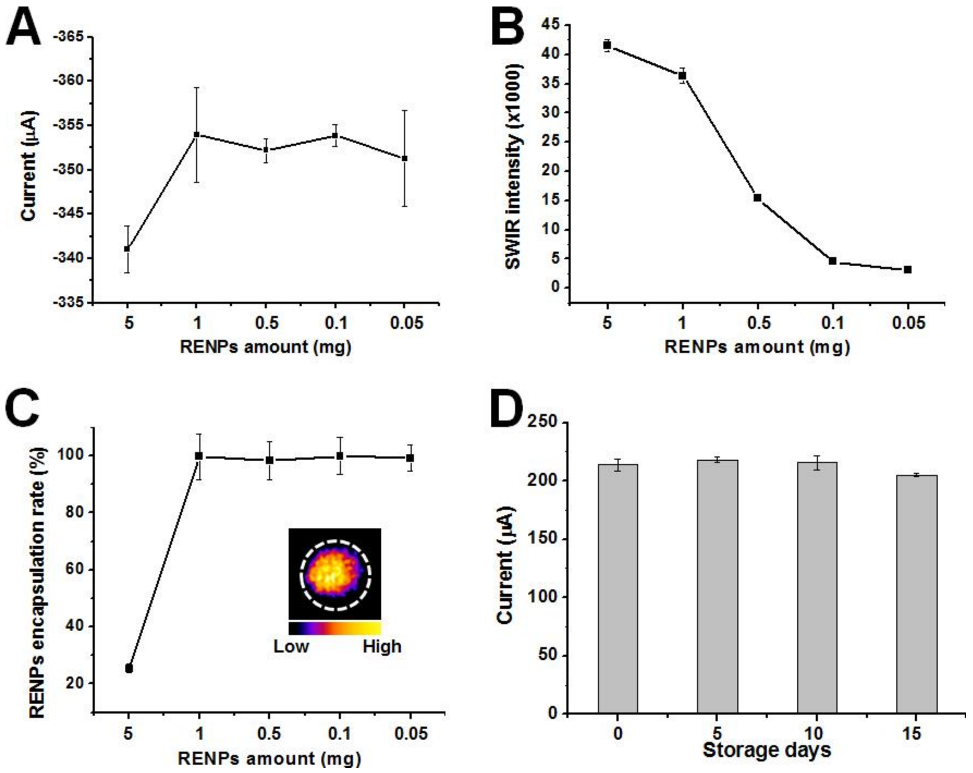

Supplementary Figure 5. Optimization of electrochemical and luminescent properties of tri-channel platform. SWV current A), SWIR luminescence intensity change B) and RENPs encapsulation rate C) as a function of RENPs amount in Cu-ALG gel. D) The stability of tri-channel platform using electrochemical signal as indicator within 15 days for storage. Data are represented as mean ± SD (n = 3)

It was found that as RENPs amount in gel increased, both current and SWIR luminescence intensity increased accordingly. However, the signal difference between 1 mg and 5 mg was quite small, which may be a result of low encapsulation rate of RENPs in gel when RENPs amount was higher than 1 mg. Therefore, we supposed that the 1 mg RENPs was close to saturated encapsulation amount in gel on electrode. Furthermore, the as-constructed tri-channel platform had outstanding stability for long-term storage, which benefitted the actual biomedical practice.

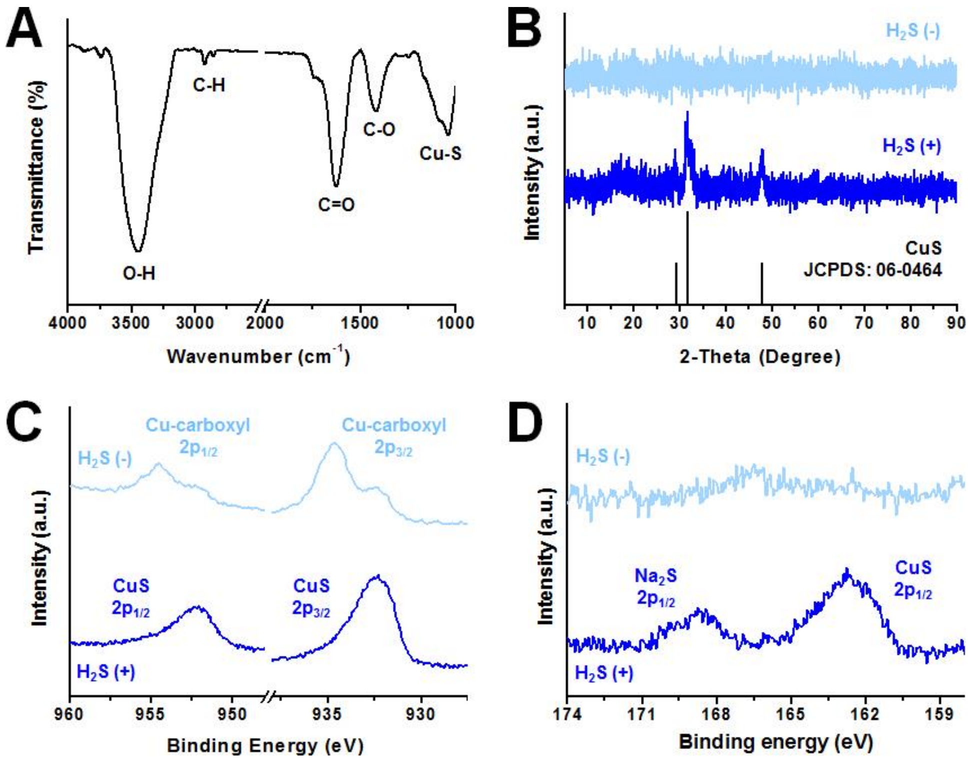

Supplementary Figure 6. Confirmation of the formation of CuS *in situ*. A) Föurier transform infrared spectrum of Cu-ALG gel after H_2S treatment. Powder X-ray diffraction patterns B), Cu_{2p} X-ray photoelectron spectra C) and S_{2p} X-ray photoelectron spectra D) of Cu-ALG gel with and without H_2S treatment.

The characteristic peak at around 1100 cm^{-1} in Föurier transform infrared spectrum of H_2S-treated gel was corresponded to the stretching vibration of Cu-S. The crystal phase of formed CuS was identified by powder X-ray diffraction patterns, corresponding to the standard cards of CuS (JCPDS: 06-0464). The energy peak at 932.32 eV and 952.17 eV in Cu_{2p} X-ray photoelectron spectra also confirmed the existence of CuS in gel after H_2S treatment, as well as the peak at 162.63 eV in S_{2p} X-ray photoelectron spectra.

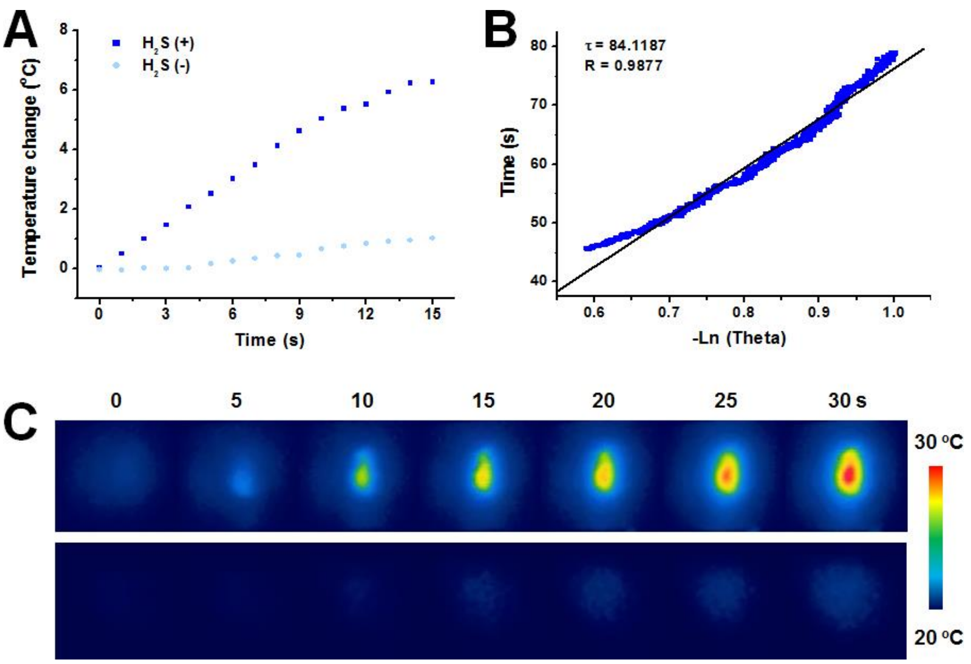

Supplementary Figure 7. H_2S-activated photothermal capacities of RENPs-ALG-Cu gel under single 808 nm laser irradiation. A) Temperature curves of gel in the presence and absence of H_2S. B) Linear time data *vs* –Ln (Theta) obtained from the cooling period. C) Photothermal images of gel in the presence (upper) and absence of H_2S (bottom) under single 808 nm laser irradiation within 30 s.

As shown in temperature curves and photothermal images, the temperature of H_2S-treated gel under laser irradiation significantly increased, which contributed to the non-irradiative relaxation and localized surface plasmon resonance of *in situ* formed CuS semiconductor. The photothermal conversion efficiency of gel under 808 nm was calculated to be 18.1% by a modified formatting of total energy conservation equation described in previous literature:

$$\eta = \frac{hS\ (T_{max} - T_{amb}) - Q_B}{I\ (1 - 10^{-\lambda_{808}})} \qquad (3)$$

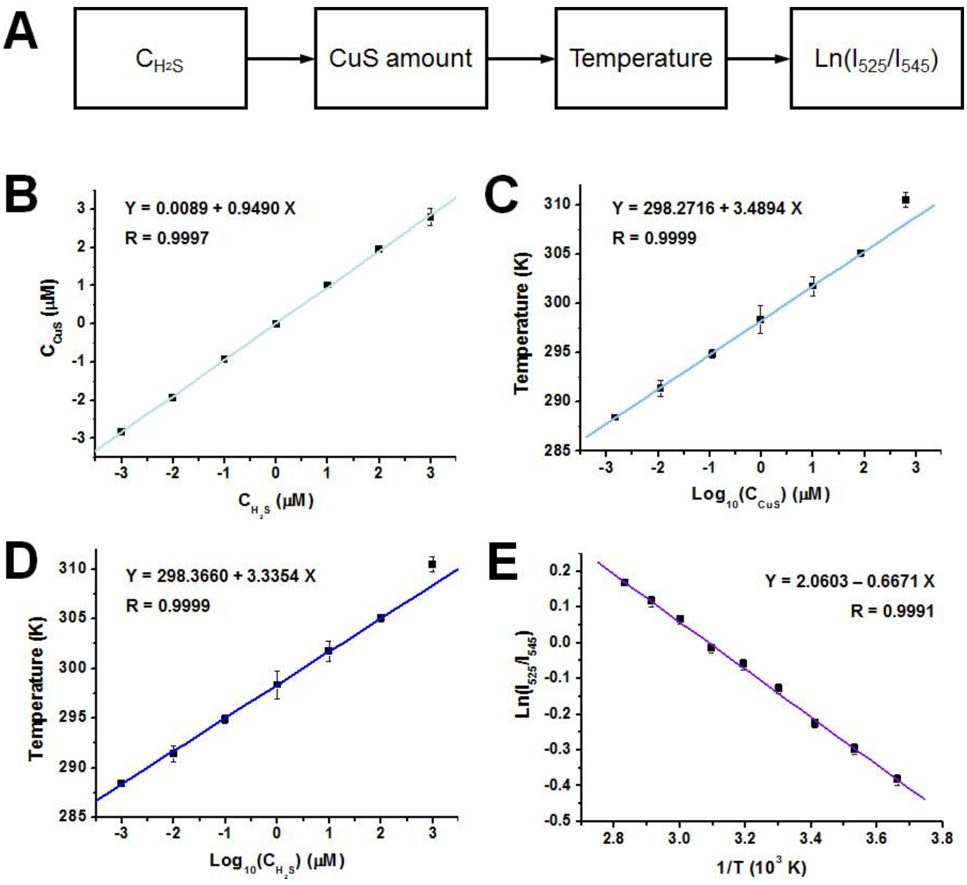

Supplementary Figure 8. Constructing relationship between C_{H_2S} and $Ln(I_{525}/I_{545})$. A) Signal transition process from C_{H_2S} to $Ln(I_{525}/I_{545})$. Linear relationship between C_{CuS} and C_{H_2S} B), temperature and $Log_{10}(C_{CuS})$ C), temperature and $Log_{10}(C_{H_2S})$ D), and $Ln(I_{525}/I_{545})$ and reciprocal of temperature (1/T) E). Through facile algebraic operation, linear relationship can be constructed between C_{H_2S} and $Ln(I_{525}/I_{545})$ of RENPs-based nanothermometer, which allows the photothermal quantification of H_2S by determining photothermal-induced temperature change of electrode *in situ*. Data are represented as mean ± SD (n = 3).

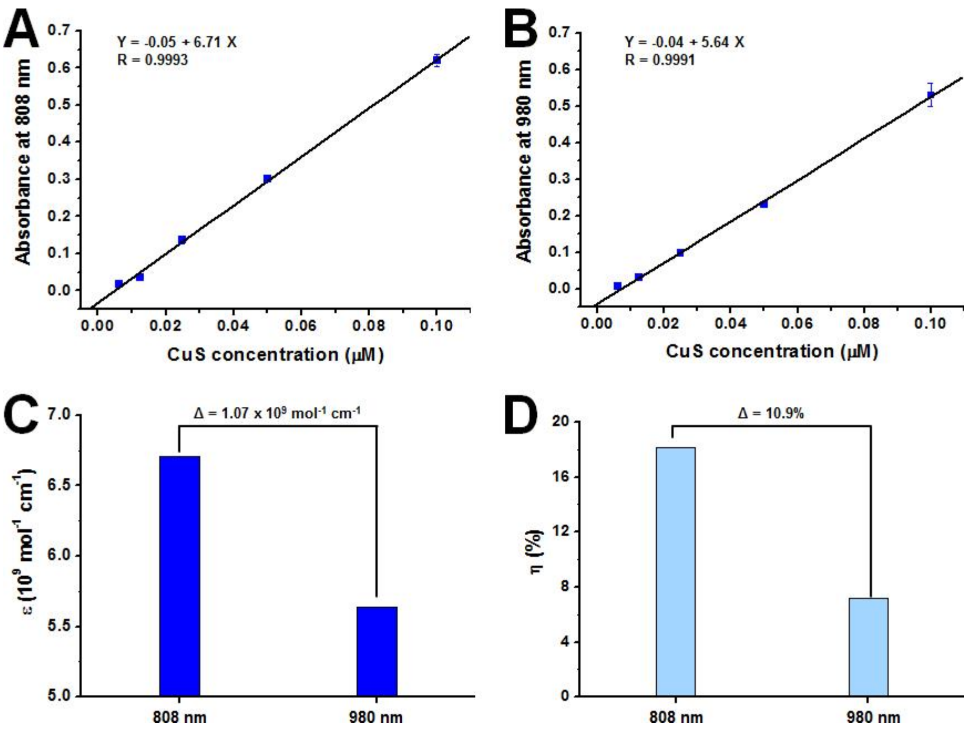

Supplementary Figure 9. Comparison of molar extinction coefficient (ε) and photothermal conversion efficiency (η) of RENPs-ALG-Cu gel at various wavelengths. Absorbance at 808 nm A) and at 980 nm B) of gel in the presence of H_2S. ε C) and η D) of H_2S-treated gel at various wavelengths. Data are represented as mean ± SD (n = 3).

It was observed that both ε and η of H_2S-treated gel at 808 nm was significantly higher than that at 980 nm ($\Delta\varepsilon = 1.07 \times 10^9$ mol^{-1} cm^{-1} and $\Delta\eta = 10.9\%$), which clearly explained the difference between 808 nm and 980 nm for generating heat.

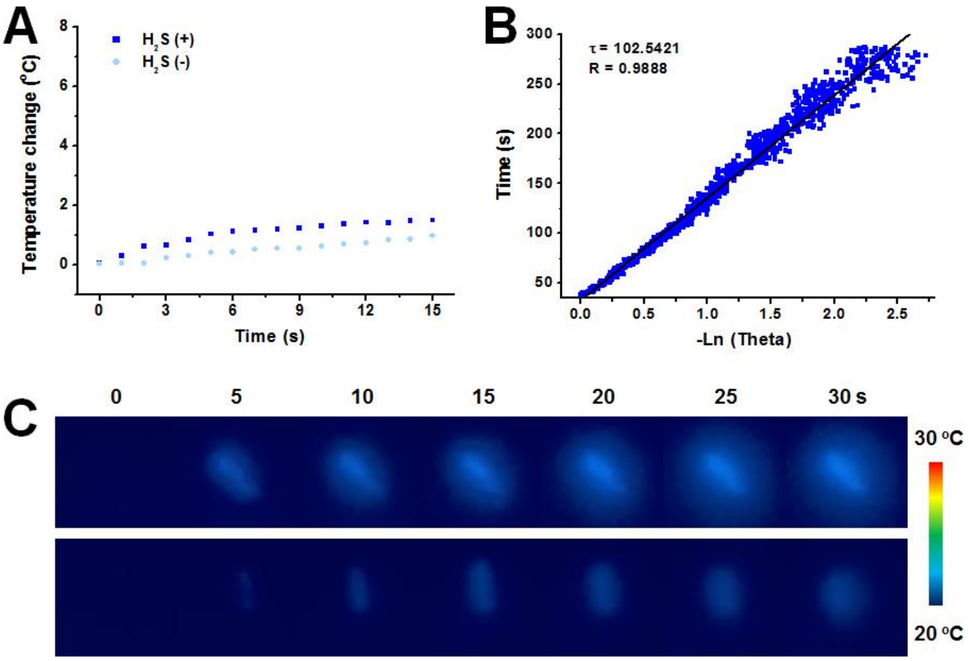

Supplementary Figure 10. H_2S-activated photothermal capacities of RENPs-ALG-Cu gel under single 980 nm laser irradiation. A) Temperature curves of gel in the presence and absence of H_2S. B) Linear time data *vs* –Ln (Theta) obtained from the cooling period. C) Photothermal images of gel in the presence (upper) and absence of H_2S (bottom) under single 980 nm laser irradiation within 30 s. The photothermal conversion efficiency of gel under 980 nm was calculated to be 7.2% (Supplementary Equation 2).

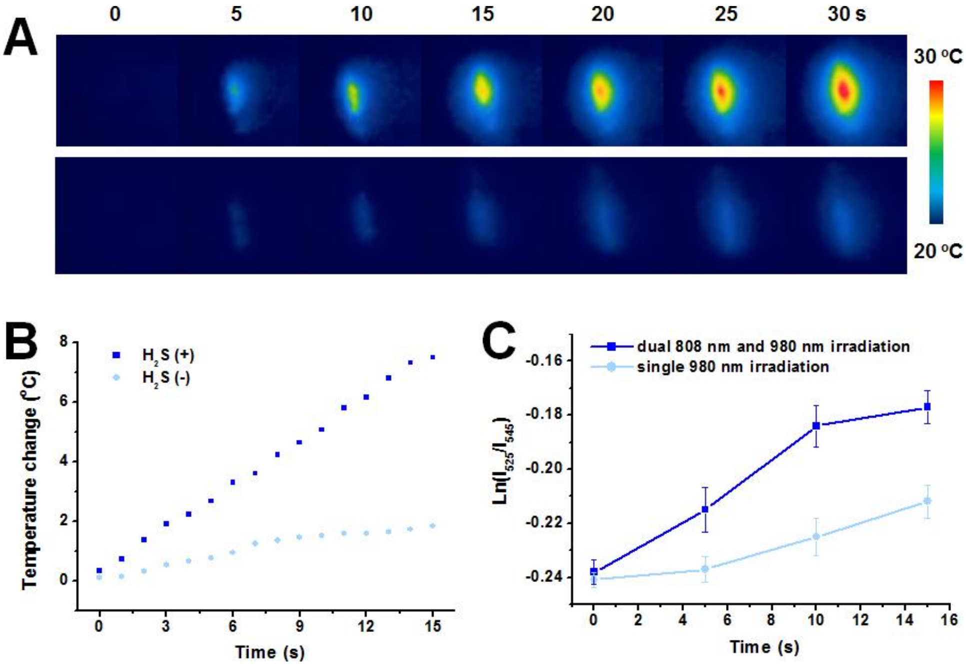

Supplementary Figure 11. H_2S-activated photothermal capacities of RENPs-ALG-Cu gel under dual 808 nm and 980 nm laser irradiation. A) Photothermal images of gel in the presence (upper) and absence of H_2S (bottom) under dual 808 nm and 980 nm laser irradiation within 30 s. B) Temperature curves of gel in the presence and absence of H_2S. C) $Ln(I_{525}/I_{545})$ curves of gel in the presence of H_2S under various laser irradiation prescriptions. Data are represented as mean ± SD (n = 3).

The time-dependent $Ln(I_{525}/I_{545})$ curves showed that the temperature of gel increase much more rapidly under dual laser irradiation, rather than single 980 nm laser irradiation, which highlighted the necessity of 808 nm laser irradiation in exciting photothermal conversion capacities of gel.

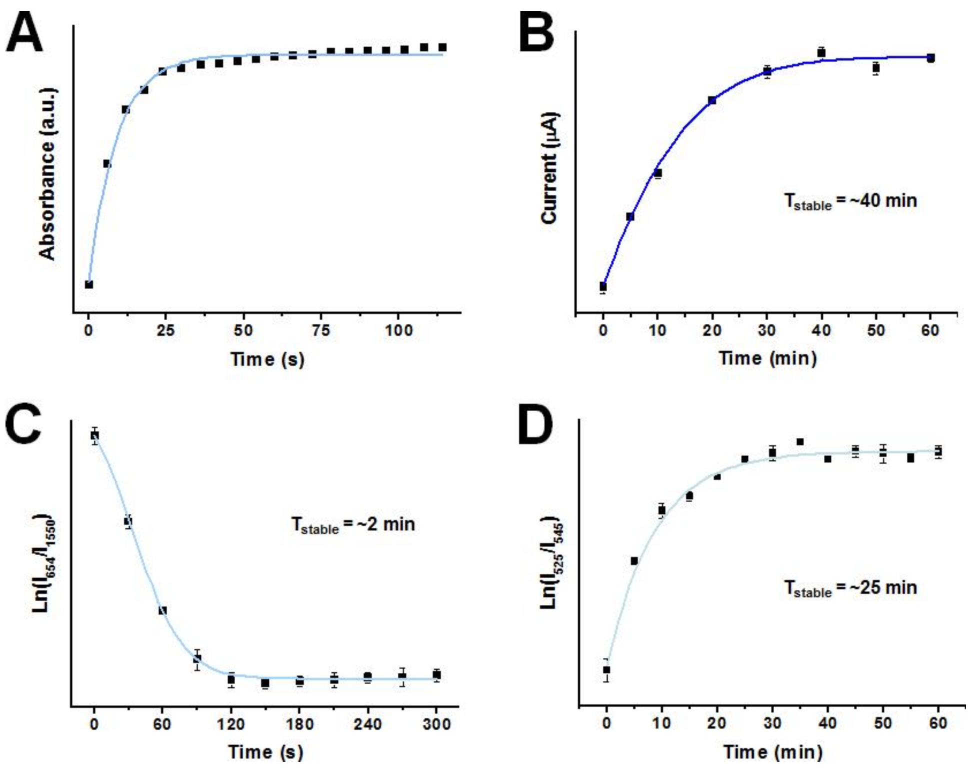

Supplementary Figure 12. Response rate of the tri-channel platform to H_2S. Absorbance at 808 nm A), current B), Ln(I_{654}/I_{1550}) C) and Ln(I_{525}/I_{545}) D) of RENPs-Cu-ALG gel in response to H_2S as a function of time. Data are represented as mean ± SD (n = 3).

Though the absorbance spectrum suggested that the reaction between H_2S and gel was rapid, a pre-incubation time longer than 40 min was appropriate to ensure the stability of all electrochemical, luminescent, and thermal signals when comprehensively considering all above results.

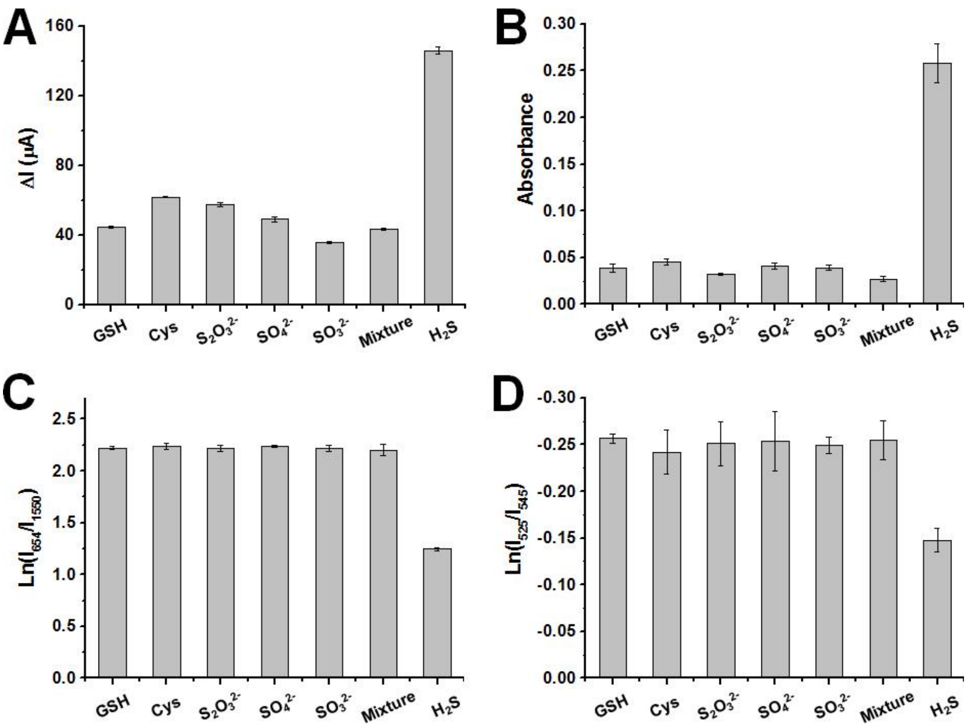

Supplementary Figure 13. Selectivity of tri-channel platform to H_2S among other biosulfurs. ΔI A), absorbance B), Ln(I_{654}/I_{1550}) C), and Ln(I_{525}/I_{545}) D) of tri-channel platform in response to H_2S (10 μM) and other biosulfurs in 100 times higher concentration (1 mM). The studied typical biosulfurs included reduced glutathione (GSH), cysteine (Cys), $S_2O_3^{2-}$, SO_4^{2-}, and SO_3^{2-}. The mixture of the above biothiols was also studied. Data are represented as mean $\pm$ SD (n = 3).

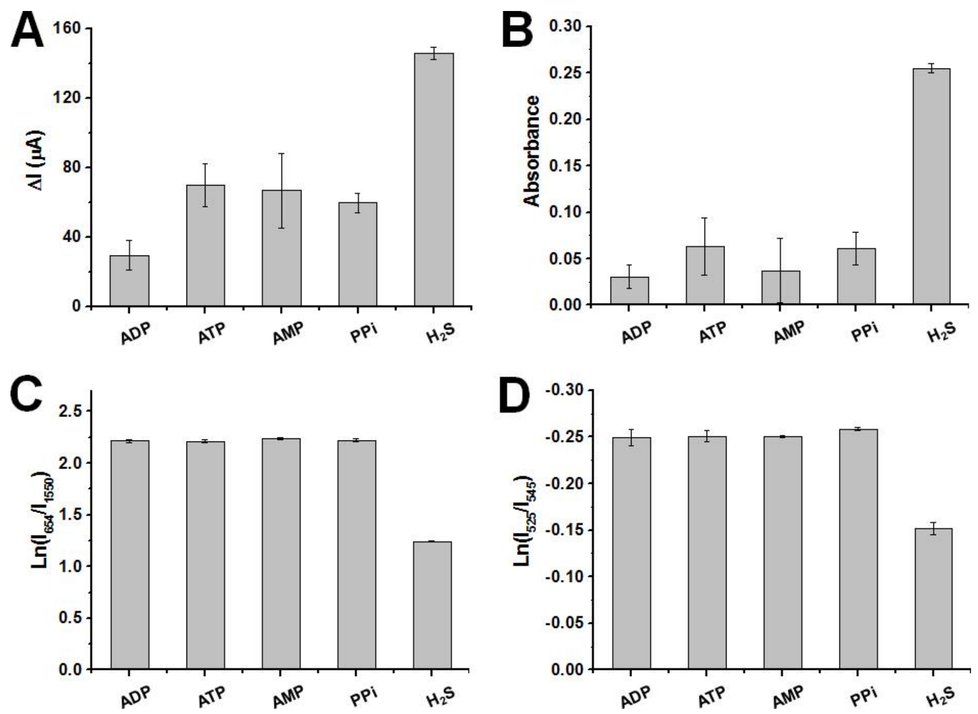

Supplementary Figure 14. Selectivity of tri-channel platform to H_2S among biophosphorus. △I A), absorbance B), Ln(I_{654}/I_{1550}) C), and Ln(I_{525}/I_{545}) D) of tri-channel platform in response to H_2S (10 μM) and other biophosphorus in 100 times higher concentration (1 mM). The studied typical biophosphorus included adenosine triphosphate (ATP), adenosine diphosphate (ADP), adenosine monophosphate (AMP) and pyrophosphoric acid (PPi). Data are represented as mean ± SD (n = 3).

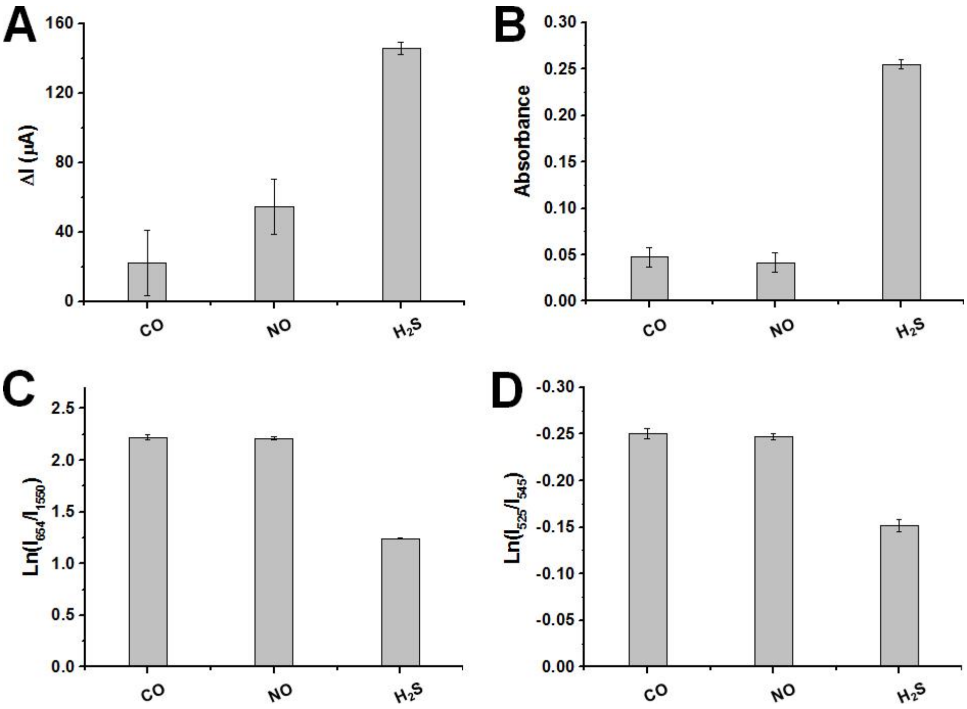

Supplementary Figure 15. Selectivity of tri-channel platform to H_2S among other cellular signal molecules. ΔI A), absorbance B), Ln(I_{654}/I_{1550}) C), and Ln(I_{525}/I_{545}) D) of tri-channel platform in response to H_2S (10 μM) and other cellular signal molecules in 100 times higher concentration (1 mM). The studied typical cellular signal molecules included NO and CO. Data are represented as mean ± SD (n = 3).

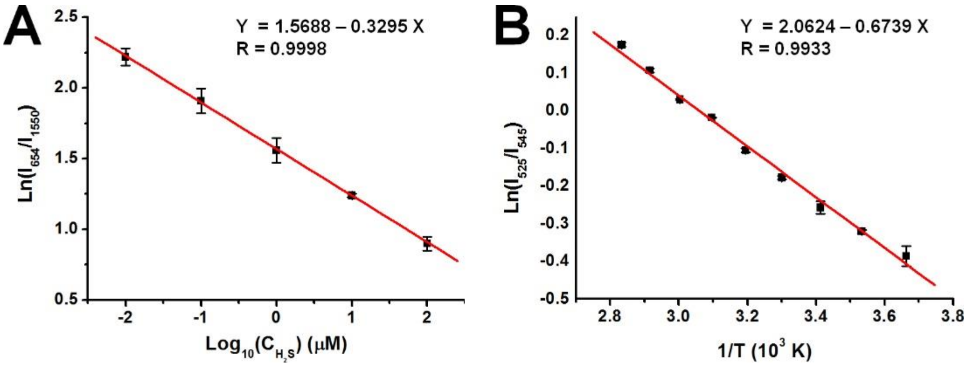

Supplementary Figure 16. Orthogonality between photothermal quantification and luminescence quantification method. A) Linear relationship between $Ln(I_{654}/I_{1550})$ of three-channel platform and various C_{H2S} in absence of 808 nm laser irradiation. B) Linear relationship between $Ln(I_{525}/I_{545})$ of three-channel platform and various temperature in presence of H_2S. Data are represented as mean ± SD (n = 3).

The linear function of luminescence quantification method in absence of 808 nm laser irradiation was similar to that in irradiation presence, while the elevated absorbance of CuS did not affect the linear relationship for temperature sensing and photothermal quantification method. The above results suggested that the luminescence and photothermal quantification method is orthogonal and would not influence the results of each other.

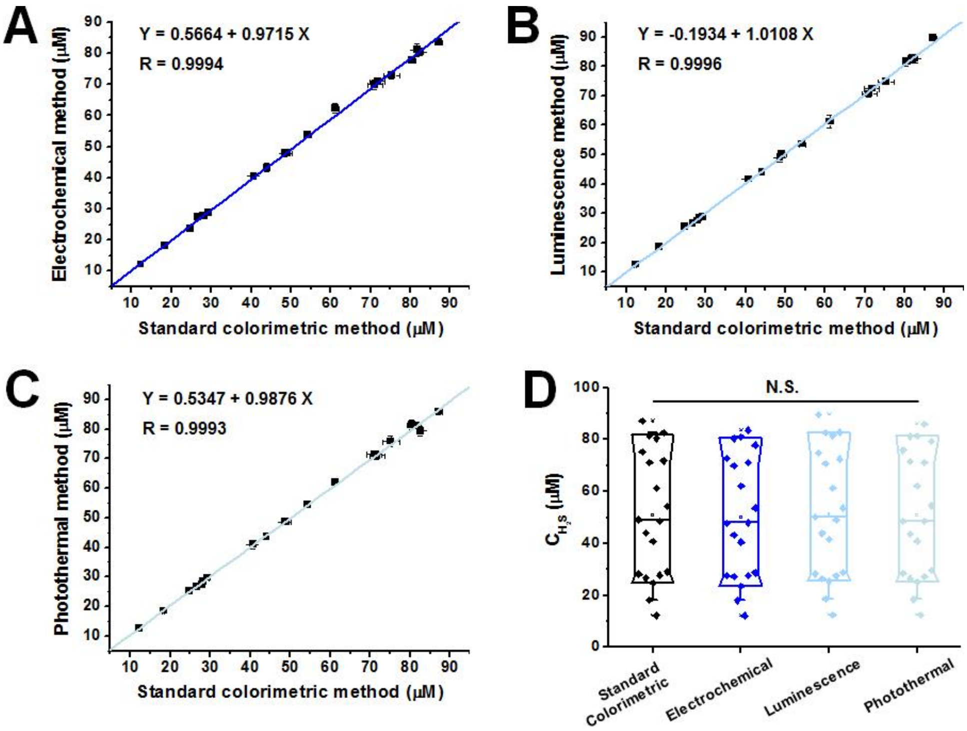

Supplementary Figure 17. Comparison between tri-signal method and standard colorimetric method for H_2S detection. C_{H_2S} determined by electrochemical method A), luminescence method B) and photothermal method C). Corresponding C_{H_2S} was previously determined by standard colorimetric method and used for comparison. D) Data statistic of the above determined C_{H_2S} by various methods. The above results illustrated that the C_{H_2S} determined by all three methods were highly in accordance with that by standard method, which suggested that all three methods were applicable for accurate quantification of H_2S. Data are represented as mean ± SD (n = 3). Statistical significance was determined from one-way t tests. N.S. means not significant.

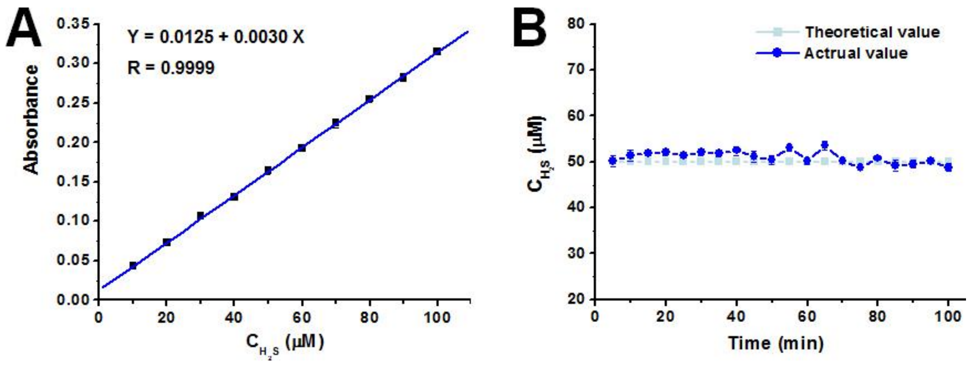

Supplementary Figure 18. C_{H_2S} uncertainty of standard H_2S solution for *in vitro* study. A) Linear relationship between various C_{H_2S} and absorbance at 665 nm that determined by typical colorimetric protocol. B) C_{H_2S} change as a function of storage time within 100 min post-preparation. Theoretical C_{H_2S} was also marked as reference. Data are represented as mean ± SD (n = 3).

It was found that the C_{H_2S} of standard H_2S solution only slightly fluctuated around 50 μM within 100 min, which suggested that the concentration of H_2S standard solution was accurate if prepared freshly and used timely. It also ensured the reliability of C_{H_2S} within incubation time of 1 h.

Supplementary Table 1. Comparison of thermal sensitivity of RENPs with other previously reported rare-earth-based luminescent nanothermometers.

**Nanothermometers**	**Thermal sensitivity (K^{-1})**	**Reference**
$NaYbF_4$:Er@$NaLuF_4$	0.0108	This work
CaF_2:Yb,Er	0.0160	Ref. 1
$NaLuF_4$:Yb,Er@$NaLuF_4$	0.0100	Ref. 2
Y_2O_3:Yb,Ho,Zn	0.0100	Ref. 3
LaF_3:Nd	0.0026	Ref. 4
CaF_2:Yb,Tm	0.0020	Ref. 1

References

1 Dong, N. N. *et al.* NIR-to-NIR two-photon excited CaF_2:Tm^{3+},Yb^{3+} nanoparticles: multifunctional nanoprobes for highly penetrating fluorescence bio-imaging. *ACS Nano.* **5**, 8665-8671, doi: 10.1021/nn202490m (20111).

2 Zhu, X. J. *et al.* Temperature-feedback upconversion nanocomposite for accurate photothermal therapy at facile temperature. *Nat. Commun.* **7**, 10437, doi:10.1038/ncomms10437 (2016).

3 Pandey, A. *et al.* Improved luminescence and temperature sensing performance of Ho^{3+}–Yb^{3+}–Zn^{2+}:Y_2O_3 phosphor. *Dalton. Trans.* **42**, 11005-11011, doi: 10.1039/C3DT50592H (2013).

4 Carrasco, E. *et al.* Intratumoral thermal reading during photo-thermal therapy by multifunctional fluorescent nanoparticles. *Adv. Funct. Mater.* **25**, 615-626, doi:10.1002/adfm.201403653 (2015).
